# Learning from the past to plan for the future: A scoping review of musculoskeletal clinical research in Sweden 2010–2020

**DOI:** 10.48101/ujms.v127.8709

**Published:** 2022-09-30

**Authors:** Elias Diarbakerli, Olof Thoreson, Martin Björklund, Leif E Dahlberg, Martin Englund, Paul Gerdhem, Joanna Kvist, Maziar Mohaddes, Anneli Peolsson, Ola Rolfson, Birgitta Öberg, Allan Abbott

**Affiliations:** 1 Department of Clinical Sciences, Intervention and Technology (CLINTEC), Karolinska Institutet, Stockholm, Sweden; 2 Department of Orthopaedics, Karolinska University Hospital, Stockholm, Sweden; 3 Department of Orthopaedics, Institute of Clinical Sciences, Sahlgrenska Academy, University of Gothenburg, and Sahlgrenska University Hospital, Gothenburg, Sweden; 4 Department of Community Medicine and Rehabilitation, Physiotherapy, Umeå University, Umeå, Sweden; 5 Centre for Musculoskeletal Research, Department of Occupational Health Sciences and Psychology, Faculty of Health and Occupational Studies, University of Gävle, Gävle, Sweden; 6 Department of Clinical Sciences Lund, Orthopaedics, Lund University, Lund, Sweden; 7 Clinical Epidemiology Unit, Orthopaedics, Department of Clinical Sciences Lund, Lund University, Lund, Sweden; 8 Stockholm Sports Trauma Research Center, Department of Molecular Medicine & Surgery, Karolinska Institute, Solna, Sweden; 9 Department of Health, Medicine and Caring Sciences, Division of Prevention, Rehabilitation and Community Medicine, Unit of Physiotherapy, Linköping University; 10 Department of Orthopaedics, Sahlgrenska University Hospital, Gothenburg, Sweden; 11 Occupational and Environmental Medicine Center, Department of Health, Medicine and Caring Sciences, Division of Prevention, Rehabilitation and Community Medicine, Linköping University, Linköping, Sweden; 12 Department of Orthopaedics, Linköping University Hospital, SE 581 83 Linköping, Sweden

**Keywords:** Musculoskeletal disorders, clinical research, research design, research funding, primary health care, secondary health care, occupational health care, Sweden

## Abstract

**Background:**

The aims of this study are to 1) determine the scope of musculoskeletal (MSK)-related clinical research in Sweden; 2) collate the amount of first-tier funding received; 3) discuss strategies and infrastructure supporting future MSK clinical trials in Sweden.

**Methods:**

A systematic scoping review protocol was applied in PubMed, Scopus, and SweCRIS databases. The articles were examined, and data were extracted in multiple stages by three blinded authors.

**Results:**

The search strategy resulted in 3,025 publications from 479 Swedish-affiliated authors. Primary health care was the basis for 14% of the publications, 84% from secondary health care, and 2% from occupational health care with a similar proportional distribution of first-tier research grant financing. Approximately one in six publications were randomized controlled trials (RCTs), while the majority were of observational cohort design. The majority of publications in primary and occupational health care were related to pain disorders (51 and 67%, respectively), especially diagnosis, prognosis, and healthcare organizational-related interventions (34%) and rehabilitation (15%) with similar proportional distribution of first-tier research grant financing. In secondary health care, rheumatic inflammatory disorder-related publications were most prevalent (30%), most frequently concerning diagnosis, prognosis, and healthcare organizational-related interventions (20%), attracting approximately half of all first-tier funding. Publications related to degenerative joint disorders (25%), fractures (16%), and joint, tendon, and muscle injuries (13%) frequently concerned surgical and other orthopedic-related interventions (16, 6, and 8%, respectively). Pain disorder-related publications (10%) as well as bone health and osteoporosis-related publications (4%) most frequently concerned diagnosis, prognosis, and healthcare organizational-related interventions (5 and 3%, respectively).

**Conclusions:**

Swedish-affiliated MSK disorder research 2010–2020 was predominantly observational cohort rather than RCT based. There was skewed first-tier funding allocation considering prevalence/incidence and burden of disease. Use of infrastructure supporting register-based RCTs, placebo-controlled RCTs, and hybrid effectiveness-implementation studies on prevention and clinical intervention is important strategies for the future in all healthcare sectors.

## Introduction

Epidemiological research on the global burden of disease between 1990 and 2019 has displayed a lowering of disease mortality rates but an increasing prevalence of disabling morbidity (1). Musculoskeletal (MSK) disorders involving all diseases and injuries related to the connective tissues of the locomotor system are among the most common cause of years lived with disability in Sweden and worldwide (1). MSK disorders affect a large proportion of the population, with work absenteeism affecting 40%, resulting in an economic cost of approximately 2% of the annual gross national product in EU member states (2). Ageing population trends and lifestyle factors are significant contributors to MSK disorders causing major burden for public health globally (3). MSK disorders are also linked to increased prevalence of pain and mental health disorders, muscle and bone mass loss, obesity, insulin resistance, risk of falls and fracture, and longer rehabilitation following injury or surgery (3).

Optimizing MSK health and wellbeing is considered key aims for the prevention and treatment of MSK disorders (4). MSK disorders are one of most common reasons for accessing primary health care in Sweden (5, 6). Many clinicians in primary care do not feel confident to diagnose and manage patients with MSK disorders, unnecessarily refer to secondary healthcare services, are not aware of recent evidence for effectiveness of different treatments, rely on shared beliefs and personal opinion rather than research evidence, or poorly adhere to existing guidelines (7–9). The result is a discordance between what is known to be effective from clinical research and what is done in clinical practice, i.e. a substantial knowledge-action gap (10, 11). Furthermore, only an estimated 14% of research knowledge is used toward improvement of health care and patient outcomes with an average delay of 17 years (10, 12). This gap between scientific knowledge and practice is estimated to result in high national spending on research without clear knowledge-action plans and continued use of low-value or ineffective care that may be insufficient or redundant, and even harmful to patients (10). To meet such challenges, building capacity and infrastructure through developing a national MSK clinical trial network in Sweden (SweMSK) may help improve the quality of future MSK research and reduce knowledge-action gaps (13).

In a first step in developing the SweMSK network and to understand the current knowledge-action gaps in Swedish MSK-related research, the following systematic scoping review aims to: 1) identify recently published (2018–2020) Swedish-affiliated MSK researchers and the scope of their MSK-related clinical research in terms of volume, design, healthcare context, types of MSK disorders, and interventions 2010–2020; 2) collate the amount of funding received for MSK disorder-related research through first-tier Swedish national competitive grants 2010–2020; 3) discuss potential strategies and infrastructure supporting future MSK-related clinical trials in Sweden.

## Materials and methods

Scoping review is a form of exploratory knowledge synthesis aimed at mapping key concepts, types of evidence, and gaps in research by systematically searching, selecting, and synthesizing existing knowledge to inform practice, policy making, and future research (14). The scoping review methodology for this study is based on a commonly used framework (15) and best praxis recommendations (16). A multidisciplinary research team with national geographic representation and expertise in the MSK disorders, epidemiology, and research synthesis was formed for the study. Reporting follows the PRISMA extension for scoping reviews (17). This scoping review used a two-step process. The first phase was to identify recently published Swedish MSK researchers through a systematic search in multiple databases. The included researchers who completed published research between 2010 and 2020 were then analyzed according to the eligibility criteria.

### Research question

#### Population/problem

All MSK studies published in 2010–2020 by recently published (2018–2020) Swedish MSK-affiliated researchers.

#### Concept

Musculoskeletal conditions – intervention studies (method, material, scope, and funding).

#### Context

What is the scope of publications and amount of first-tier national competitive funding? And based on this, what are potential strategies and infrastructure supporting future MSK-related clinical trials in Sweden?

### Identification of relevant studies

#### Eligibility

Inclusion criteria were MSK disorder-related clinical trials, and observational studies and qualitative studies published in English by recently published (2018–2020) Swedish-affiliated researchers. The reasoning for limiting to recent publications was to aid identifying currently active researchers for the development of the SweMSK network. Based on this cohort of recently published authors, the search was expanded to capture each individual author’s scope of MSK-related publications 2010–2020. Phase 1 clinical trials, reviews, study protocols, odontology studies, and studies not fully meeting the inclusion criteria were excluded.

#### Information sources and search strategy

In a first step, the following search strategies were conducted:

1. Research article publication databases

PubMed search – Search terms were applied to the title and abstract of PubMed indexed publications. Using the Boolean operator ‘AND’, Swedish affiliation was added to MSK disorder-related search terms for different bodily locations which were bundled using the Boolean operator ‘OR’. After screening resulting titles, the research team refined the MSK disorder-related search terms. Furthermore, the Boolean operator ‘NOT’ was used to improve sensitivity of the search by filtering out non-MSK disorder-related studies. The truncation symbol * was used for search terms that have a similar root. Due to the large number of results, filters for randomized controlled trial (RCT), clinical trial, and observational study were applied. The specific search strategy is outlined in Appendix 1.

Scopus search – Search terms were applied to the title, abstract, and keywords of Scopus indexed publications. MSK symptom and diagnosis-related search terms for different bodily locations were combined using the Boolean operator ‘OR’ and restricted to Swedish affiliation using the Boolean operator ‘AND’. After screening resulting titles, the Boolean operator ‘AND NOT’ was used to improve sensitivity of the search by filtering out non-MSK disorder-related studies. The truncation symbol * was used for search terms that have a similar root. Limitations to articles published in scientific journals in the subject areas ‘Medicine’ and ‘Health professions’ were applied. No filters were used for specific research designs as a sensitivity measure in the case that research design filter posed on the PubMed search had missed studies. The specific search strategy is outlined in Appendix 1.

2. Research funding database – SweCRIS

SweCRIS is a national database that outlines how participating research funding bodies have distributed their money to Swedish recipients. The research funding bodies that supply SweCRIS with data are first-tier funding bodies, either state or privately financed and open for national competitive funding rounds. The included research funders providing financing for medical and health care focused research are the Swedish Research Council (VR), Swedish Research Council for Health, Working Life and Welfare (FORTE), VINNOVA, Swedish Foundation for Strategic Research (SSF), and yearly updates from the European Union Horizon 2020 program. Second tier research funding from company-based research fonds, regional funding bodies, and the contract between the Swedish government and seven regions concerning the financing of physician education, clinical research, and healthcare system development (18) are not included in the SweCRIS database. Information in SweCRIS is structured according to grant contracts. SweCRIS is managed by the Swedish Research Council on behalf of the Government. The database was filtered for the subject areas orthopedics, rheumatology, physiotherapy, and pain. All years from the start of the database in 2010 up to the end of 2020 were included.

### Study selection

Results from searches of the information sources were entered into an Excel file. Studies underwent independent review by either authors ED, OT, or AA using the above-named eligibility criteria, and any ambiguities were resolved by consensus between reviewers.

### Charting the data from the search results

For each of the included studies, the following data were extracted:

1. Study characteristics: Journal reference.
2. Study design: 1 = Randomized controlled trial, 2 = Case–control study, 3 = Cohort study, 4 = Qualitative study.
3. Symptom location: 1 = Temporomandibular joint (TMJ) and head, 2 = Back, neck, and pelvis, 3 = Upper limb, 4 = Lower limb, 5 = Multisite.
4. Diagnostic category: 1 = Pain disorders, 2 = Joint, tendon, and muscle injuries, 3 = Degenerative joint disorders, 4 = Rheumatic inflammatory disorders, 5 = Bone health and osteoporosis, 6 = Fractures, 7 = Other
5. Health care context: 1 = Primary health care, 2 = Secondary health care, 3 = Occupational health care.
6. Intervention: 1 = Diagnosis, prognosis, and healthcare organizational related, 2 = Prevention, 3 = Rehabilitation, 4 = Environmental modification, Activities of daily living (ADL) aids, and physical modalities, 5 = Pharmacological, 6 = Surgical and other orthopedic-related interventions.

Data extraction from included studies was independently performed by one reviewer using a standardized data extraction form and afterward cross-checked by an additional reviewer. Ambiguities were resolved afterward through discussion between reviewers.

### Collating and summarizing results

Descriptive statistics including frequencies, proportions, and total sums were performed for the above stated data in IBM SPSS Statistics version 25.

## Results

**Figure 1:**
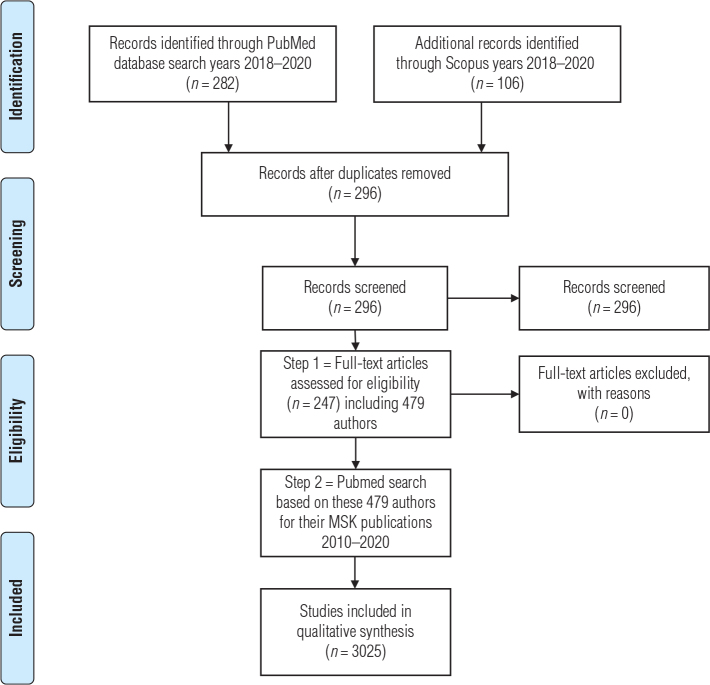
PRISMA flow chart.

Initially, 282 publications from PubMed and 106 publications from Scopus databases were identified between the years 2018 and 2020. After screening, 296 of these publications remained after removal of duplicates, and 247 remained after removal of studies not fulfilling eligibility criteria. From these studies, 479 Swedish-affiliated authors were identified. A PubMed search based on these 479 authors for their MSK publications 2010–2020 resulted in 3,025 publications. A PRISMA flow chart is presented in Figure 1. The distribution of publication based on healthcare context was 412/3,025 (14%) from primary care, 2,552/3,025 (84%) from secondary care, and 60/3,025 (2%) from occupational healthcare system.

### Primary healthcare publications

**Figure 2:**
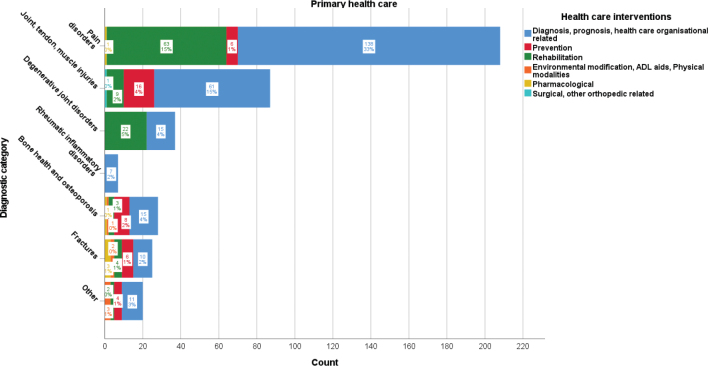
Frequency and proportion of primary healthcare intervention categories per diagnostic category for musculoskeletal disorder-related Swedish research publications 2010–2020.

The distribution of MSK disorder publications based on primary healthcare system interventions is presented in Figure 2. Pain disorder-related publications were most prevalent (208/412, 51%) and mainly concerned diagnosis, prognosis, and healthcare organizational-related interventions (138/412, 34%) and rehabilitation (63/412, 15%). Joint, tendon, and muscle injury-related publications (87/412, 21%) mainly concerned diagnosis, prognosis, and healthcare organizational-related interventions (61/412, 15%). Degenerative joint disorder-related publications (37/412, 9%) mainly concerned rehabilitation (22/412, 5%). Bone health and osteoporosis-related publications (28/412, 7%) mainly concerned diagnosis, prognosis, and healthcare organizational-related interventions (15/412, 4%). Fracture, rheumatic inflammatory, and other-related publications together (52/412, 13%) mainly concerned diagnosis, prognosis, and healthcare organizational-related interventions (28/412, 7%).

The frequencies of publications of RCT design (86/412, 21%), case–control studies (43/412, 10%), cohort studies (260/412, 63%), and qualitative studies (23/412, 6%) are displayed in Table 1. RCT design was most frequent for studies that were related to rehabilitative interventions (56/412, 14%), especially regarding pain disorders (44/412, 11%) located to the back, neck, or pelvis (32/412, 8%). Cohort studies were most frequent for publications concerning diagnosis, prognosis, and healthcare organizational-related interventions (205/412, 50%), especially regarding pain disorders (94/412, 23%) located to the back, neck, or pelvis (57/412, 14%). Case–control and qualitative studies followed a similar trend.

**Table 1:** Frequency of study designs for musculoskeletal disorder-related Swedish research publications 2010–2020 based on primary healthcare intervention.

Diagnostic category		Diagnosis, prognosis, and healthcare organizational-related interventions				Preventative interventions				Rehabilitation interventions				Environmental modification, ADL aids and physical modalities				Pharmacological interventions				Surgery and other orthopedic-related interventions				Sub total	Total
		RCT	CC	C	Q	RCT	CC	C	Q	RCT	CC	C	Q	RCT	CC	C	Q	RCT	CC	C	Q	RCT	CC	C	Q		
Pain disorders	TMJ, head	0	8	1	1	0	0	0	0	4	0	0	0	0	0	0	0	0	0	0	0	0	0	0	0	14	208
	Back, neck, and pelvis	4	12	57	3	0	0	5	0	32	2	10	1	0	0	0	0	0	0	0	0	0	0	0	0	126	
	Upper limb	0	0	4	0	0	0	0	0	0	0	0	1	0	0	0	0	0	0	0	0	0	0	0	0	5	
	Lower limb	0	0	0	1	0	0	0	0	0	0	0	0	0	0	0	0	0	0	0	0	0	0	0	0	1	
	Multisite	2	4	36	5	1	0	0	0	8	1	4	0	0	0	0	0	0	0	1	0	0	0	0	0	62	
Joint, tendon, and muscle injuries	TMJ, head	0	0	0	0	0	0	0	0	0	0	0	0	0	0	0	0	0	0	0	0	0	0	0	0	0	87
	Back, neck, and pelvis	0	0	2	0	0	0	0	0	0	0	0	0	0	0	0	0	0	0	0	0	0	0	0	0	2	
	Upper limb	0	0	2	0	0	0	0	0	2	1	0	0	0	0	0	0	0	0	0	0	1	0	0	0	6	
	Lower limb	0	2	25	0	4	1	2	1	0	0	4	1	0	0	0	0	0	0	0	0	0	0	0	0	40	
	Multisite	0	0	29	1	5	2	1	0	0	0	1	0	0	0	0	0	0	0	0	0	0	0	0	0	39	
Degenerative joint disease	TMJ, head	0	0	0	0	0	0	0	0	0	0	0	0	0	0	0	0	0	0	0	0	0	0	0	0	0	37
	Back, neck, and pelvis	0	0	0	0	0	0	0	0	2	0	0	0	0	0	0	0	0	0	0	0	0	0	0	0	2	
	Upper limb	0	0	1	0	0	0	0	0	0	0	0	0	0	0	0	0	0	0	0	0	0	0	0	0	1	
	Lower limb	2	1	6	0	0	0	0	0	1	2	13	3	0	0	0	0	0	0	0	0	0	0	0	0	28	
	Multisite	0	0	5	0	0	0	0	0	0	0	1	0	0	0	0	0	0	0	0	0	0	0	0	0	6	
Rheumatic inflammatory disorders	TMJ, head	0	0	0	0	0	0	0	0	0	0	0	0	0	0	0	0	0	0	0	0	0	0	0	0	0	7
	Back, neck, and pelvis	0	0	0	1	0	0	0	0	0	0	0	0	0	0	0	0	0	0	0	0	0	0	0	0	1	
	Upper limb	0	0	0	0	0	0	0	0	0	0	0	0	0	0	0	0	0	0	0	0	0	0	0	0	0	
	Lower limb	0	0	0	0	0	0	0	0	0	0	0	0	0	0	0	0	0	0	0	0	0	0	0	0	0	
	Multisite	0	0	6	0	0	0	0	0	0	0	0	0	0	0	0	0	0	0	0	0	0	0	0	0	6	
Bone health and osteoporosis	TMJ, head	0	0	0	0	0	0	0	0	0	0	0	0	0	0	0	0	0	0	0	0	0	0	0	0	0	28
	Back, neck, and pelvis	0	0	0	0	0	0	0	0	2	0	0	0	0	0	0	1	0	0	0	0	0	0	0	0	3	
	Upper limb	0	0	0	0	0	0	0	0	0	0	0	0	0	0	0	0	0	0	0	0	0	0	0	0	0	
	Lower limb	0	0	2	0	0	0	0	0	0	0	0	0	0	0	0	0	0	0	0	0	0	0	0	0	2	
	Multisite	1	0	11	1	3	2	3	0	0	0	1	0	0	0	0	0	0	0	1	0	0	0	0	0	23	
Fracture	TMJ, headache	0	0	0	0	0	0	0	0	0	0	0	0	0	0	0	0	0	0	0	0	0	0	0	0	0	25
	Back, neck, and pelvis	0	0	0	0	0	0	0	0	0	0	0	0	0	0	0	0	0	0	0	0	0	0	0	0	0	
	Upper limb	0	0	0	0	0	0	0	0	0	0	0	0	0	0	0	0	0	0	0	0	0	0	0	0	0	
	Lower limb	0	0	2	0	0	0	1	0	4	0	0	0	0	0	1	0	0	1	1	0	0	0	0	0	10	
	Multisite	0	1	7	0	0	3	2	0	0	0	0	0	0	0	1	0	0	0	1	0	0	0	0	0	15	
Other	TMJ, headache	0	0	0	0	0	0	0	0	0	0	0	0	0	0	0	0	0	0	0	0	0	0	0	0	0	20
	Back, neck, and pelvis	0	0	0	0	0	0	0	0	0	0	0	0	0	0	0	0	0	0	0	0	0	0	0	0	0	
	Upper limb	0	0	0	0	0	0	0	0	0	0	0	0	0	0	0	0	0	0	0	0	0	0	0	0	0	
	Lower limb	0	0	0	0	0	0	0	0	0	0	0	0	0	0	0	0	0	0	0	0	0	0	0	0	0	
	Multisite	1	0	9	1	3	0	1	0	1	0	0	1	3	0	0	0	0	0	0	0	0	0	0	0	20	
	Subtotal	10	28	205	14	16	8	15	1	56	6	34	7	3	0	2	1	0	1	4	0	1	0	0	0		
	Total	257				40				103				6				5				1					412

RCT = Randomized controlled trial, CC = Case–control study, C = Cohort study, Q = Qualitative study, TMJ = Temporomandibular joint.

### Secondary healthcare publications

**Figure 3:**
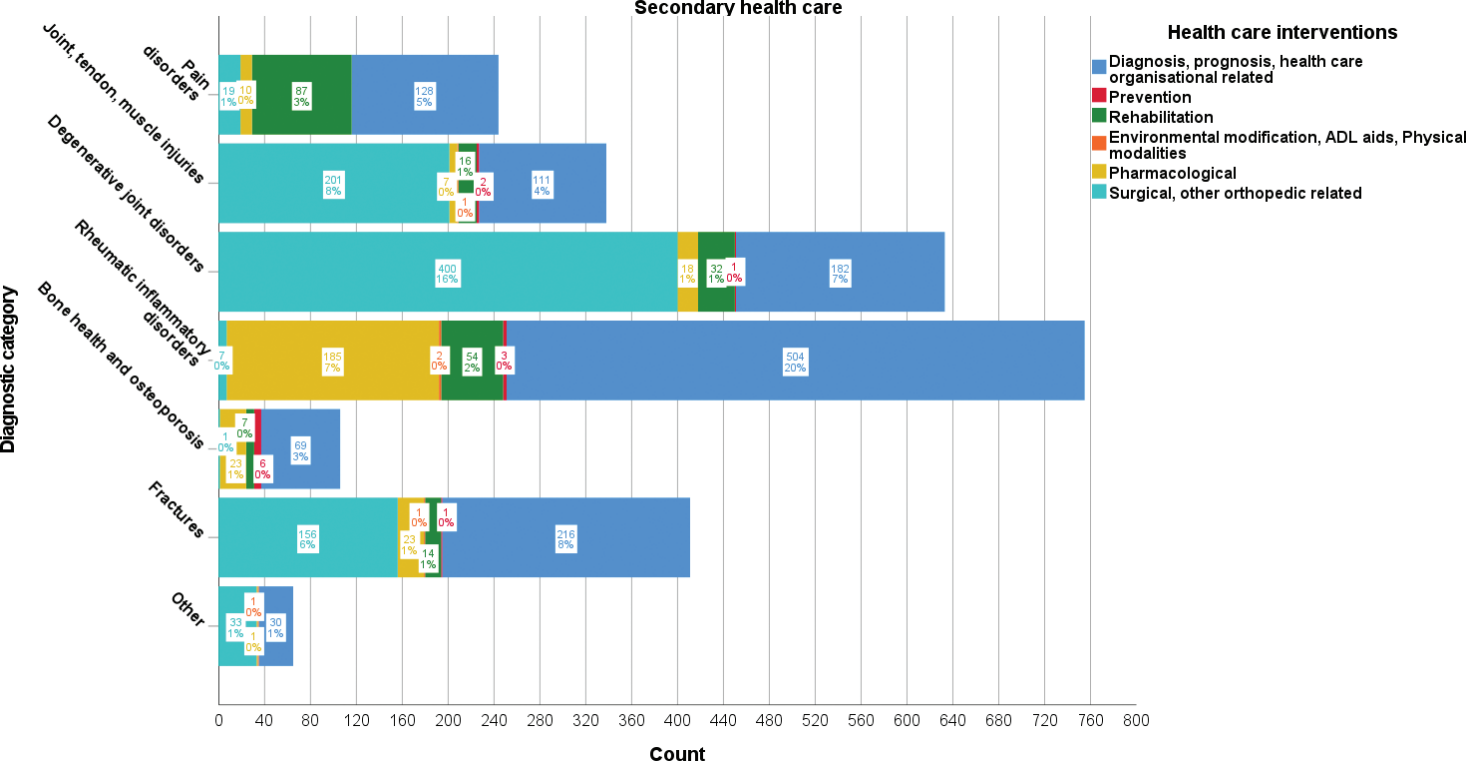
Frequency and proportion of secondary healthcare intervention categories per diagnostic category for musculoskeletal disorder-related Swedish research publications 2010–2020.

The distribution of MSK disorder publications based on secondary healthcare system interventions is presented in Figure 3. Rheumatic inflammatory disorder-related publications were most prevalent (755/2,552, 30%), frequently concerning diagnosis, prognosis, and healthcare organizational-related interventions (504/2,552, 20%), pharmacological interventions (185/2,552, 7%), and rehabilitation (54/2,552, 2%). Publications related to degenerative joint disorders (633/2,552, 25%), fractures (411/2,552, 16%), and joint, tendon, and muscle injuries (338/2,552, 13%) frequently concerned surgical and other orthopedic-related interventions (400/2,552, 16%; 156/2,552, 6%; 201/2,552, 8%) as well as diagnosis, prognosis, and healthcare organizational-related interventions (182/2,552, 7%; 216/2,552, 8%; 111/2,552, 4%), respectively. Pain disorder-related publications (244/2,552, 10%) most frequently concerned diagnosis, prognosis, and healthcare organizational-related interventions (128/2,552, 5%) and rehabilitation interventions (87/2,552, 3%). Bone health and osteoporosis-related publications 106/2,552 (4%) most frequently concerned diagnosis, prognosis, and healthcare-related interventions (69/2,552, 3%) and pharmacological interventions (23/2,552, 1%).

The frequency of publications of RCT design (381/2,552, 15%), case–control studies (373/2,552, 15%), cohort studies (1,720/2,552, 67%), and qualitative studies (78/2,552, 3%) are displayed in Table 2. RCT studies were most frequently concerning surgical and other orthopedic-related interventions (179/2,552, 7%), pharmacological interventions (91/2,552, 4%), and rehabilitation (80/2,552, 3%). Cohort studies were frequently concerning diagnosis, prognosis, and healthcare organizational-related studies (887/2,552, 35%) and surgical and other orthopedic-related interventions (584/2,552, 23%), pharmacological interventions (154/2,552, 6%), and rehabilitation (88/2,552, 3%). Case–controls and qualitative studies followed a similar trend.

**Table 2:** Frequency of study designs for musculoskeletal disorder-related Swedish research publications 2010–2020 based on secondary healthcare intervention.

Diagnostic category		Diagnosis, prognosis, and healthcare organizational-related interventions				Preventative interventions				Rehabilitation interventions				Environmental modification, ADL aids, and physical modalities				Pharmacological interventions				Surgery and other orthopedic-related interventions				Sub total	Total
		RCT	CC	C	Q	RCT	CC	C	Q	RCT	CC	C	Q	RCT	CC	C	Q	RCT	CC	C	Q	RCT	CC	C	Q		
Pain disorders	TMJ, head	0	6	0	0	0	0	0	0	1	1	1	0	0	0	0	0	1	0	0	0	0	0	0	0	10	244
	Back, neck, and pelvis	0	23	16	0	0	0	0	0	10	0	10	1	0	0	0	0	1	0	3	0	0	0	12	0	76	
	Upper limb	0	2	1	0	0	0	0	0	4	1	2	0	0	0	0	0	0	0	0	0	1	0	3	0	14	
	Lower limb	0	3	3	0	0	0	0	0	0	0	0	0	0	0	0	0	1	0	0	0	0	0	3	0	10	
	Multisite	1	35	36	2	0	0	0	0	17	5	24	10	0	0	0	0	3	0	1	0	0	0	0	0	134	
Joint, tendon, and muscle injuries	TMJ, head	0	0	0	0	0	0	0	0	0	0	0	0	0	0	0	0	0	0	0	0	0	0	0	0	0	338
	Back, neck, and pelvis	0	0	1	0	0	0	0	0	0	0	0	0	0	0	0	0	0	0	0	0	1	0	0	0	2	
	Upper limb	0	0	8	3	0	0	0	0	1	0	0	0	0	0	0	0	3	0	1	0	6	0	18	0	40	
	Lower limb	1	16	77	1	0	0	2	0	5	0	10	0	0	0	1	0	2	0	1	0	44	13	119	0	292	
	Multisite	0	0	4	0	0	0	0	0	0	0	0	0	0	0	0	0	0	0	0	0	0	0	0	0	4	
Degenerative joint disease	TMJ, head	0	0	0	0	0	0	0	0	0	0	0	0	0	0	0	0	0	0	0	0	0	0	0	0	0	633
	Back, neck, and pelvis	0	6	24	1	0	0	0	1	6	1	7	2	0	0	0	0	0	0	1	0	23	5	59	0	136	
	Upper limb	1	2	9	0	0	0	0	0	0	0	0	0	0	0	0	0	0	0	0	0	3	0	11	0	26	
	Lower limb	3	33	93	1	0	0	0	0	10	3	3	0	0	0	0	0	11	0	4	0	59	10	223	7	460	
	Multisite	0	2	5	2	0	0	0	0	0	0	0	0	0	0	0	0	1	0	1	0	0	0	0	0	11	
Rheumatic inflammatory disorders	TMJ, head	0	0	0	0	0	0	0	0	0	0	0	0	0	0	0	0	0	0	0	0	0	0	0	0	0	755
	Back, neck, and pelvis	0	8	31	1	0	0	0	0	1	0	2	0	0	0	0	0	1	0	10	0	0	0	1	0	55	
	Upper limb	0	1	1	0	0	0	0	0	1	0	0	0	0	0	0	0	0	0	0	0	0	0	2	0	5	
	Lower limb	0	0	0	0	0	0	0	0	1	0	0	0	0	0	0	0	0	0	2	0	0	0	1	0	4	
	Multisite	13	127	306	16	2	1	0	0	10	4	27	8	2	0	0	0	52	13	103	4	0	0	3	0	691	
Bone health and osteoporosis	TMJ, head	0	0	0	0	0	0	0	0	0	0	0	0	0	0	0	0	0	0	0	0	0	0	0	0	0	106
	Back, neck, and pelvis	0	1	1	0	0	0	0	0	0	0	0	0	0	0	0	0	0	0	0	0	0	0	0	0	2	
	Upper limb	0	0	1	0	0	0	0	0	0	0	0	0	0	0	0	0	0	0	0	0	0	0	0	0	1	
	Lower limb	0	0	1	0	0	0	0	0	0	0	0	0	0	0	0	0	0	0	2	0	0	0	1	0	4	
	Multisite	0	10	55	0	1	1	4	0	5	1	0	1	0	0	0	0	4	3	14	0	0	0	0	0	99	
Fracture	TMJ, headache	0	0	0	0	0	0	0	0	0	0	0	0	0	0	0	0	0	0	0	0	0	0	0	0	0	411
	Back, neck, and pelvis	0	0	5	0	0	0	0	0	0	0	0	0	0	0	0	0	3	0	0	0	7	1	13	0	29	
	Upper limb	2	2	15	1	0	0	0	0	1	0	0	0	0	0	0	0	3	0	1	0	12	2	17	0	56	
	Lower limb	2	5	80	5	0	0	0	0	7	4	2	0	1	0	0	0	5	2	5	0	21	4	71	3	217	
	Multisite	2	6	91	0	0	0	0	1	0	0	0	0	0	0	0	0	0	0	4	0	1	0	4	0	109	
Other	TMJ, headache	0	0	0	0	0	0	0	0	0	0	0	0	0	0	0	0	0	0	0	0	0	0	0	0	0	65
	Back, neck, and pelvis	0	2	15	0	0	0	0	0	0	0	0	0	0	0	0	0	0	0	0	0	0	6	14	0	37	
	Upper limb	0	0	0	0	0	0	0	0	0	0	0	0	0	0	0	0	0	0	0	0	0	0	0	0	0	
	Lower limb	0	0	3	0	0	0	0	0	0	0	0	0	0	0	0	1	0	0	0	0	0	0	3	1	8	
	Multisite	0	1	5	4	0	0	0	0	0	0	0	0	0	0	0	0	0	0	1	0	1	1	6	1	20	
	Subtotal	25	291	887	37	3	2	6	2	80	20	88	22	3	0	1	1	91	18	154	4	179	42	584	12		
	Total	1,240				13				210				5				267				817					2,552

RCT = Randomized controlled trial, CC = Case–control study, C = Cohort study, Q = Qualitative study, TMJ = Temporomandibular joint.

### Occupational healthcare publications

**Figure 4:**
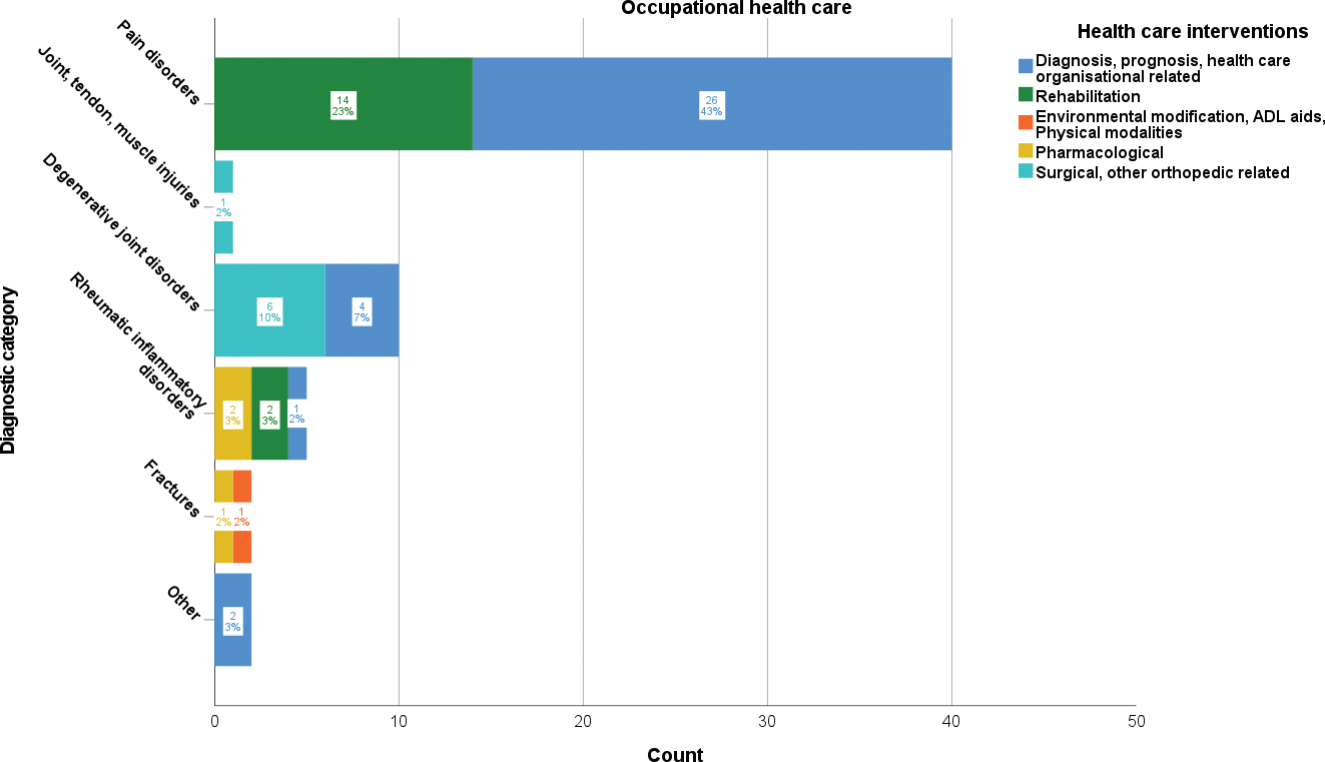
Frequency and proportion of occupational healthcare intervention categories per diagnostic category for musculoskeletal disorder-related Swedish research publications 2010–2020.

The distribution of MSK disorder publications based on occupational healthcare system interventions is presented in Figure 4. Pain disorder-related publications (40/60, 67%) most frequently concerned diagnosis, prognosis, and healthcare organizational-related interventions (26/60, 43%), followed by rehabilitation interventions (14/60, 23%). Only few other publications were related to other diagnostic groups and interventions. The frequencies of publications of RCT design (9/60, 15%), case–control studies (9/60, 15%), cohort studies (39/60, 65%), and qualitative studies (3/60, <1%) are displayed in Table 3.

**Table 3:** Frequency of study designs for musculoskeletal disorder-related Swedish research publications 2010–2020 based on occupational healthcare intervention.

Diagnostic category		Diagnosis, prognosis, and healthcare organizational-related interventions				Preventative interventions				Rehabilitation interventions				Environmental modification, ADL aids, and physical modalities				Pharmacological interventions				Surgery and other orthopedic-related interventions				Sub total	Total
		RCT	CC	C	Q	RCT	CC	C	Q	RCT	CC	C	Q	RCT	CC	C	Q	RCT	CC	C	Q	RCT	CC	C	Q		
Pain disorders	TMJ, head	0	0	0	0	0	0	0	0	0	0	0	0	0	0	0	0	0	0	0	0	0	0	0	0	0	40
	Back, neck, and pelvis	0	4	10	0	0	0	0	0	7	0	4	0	0	0	0	0	0	0	0	0	0	0	0	0	25	
	Upper limb	0	2	0	0	0	0	0	0	0	0	0	0	0	0	0	0	0	0	0	0	0	0	0	0	2	
	Lower limb	0	0	0	0	0	0	0	0	0	0	0	0	0	0	0	0	0	0	0	0	0	0	0	0	0	
	Multisite	0	1	8	1	0	0	0	0	1	0	2	0	0	0	0	0	0	0	0	0	0	0	0	0	13	
Joint, tendon, and muscle injuries	TMJ, head	0	0	0	0	0	0	0	0	0	0	0	0	0	0	0	0	0	0	0	0	0	0	0	0	0	1
	Back, neck, and pelvis	0	0	0	0	0	0	0	0	0	0	0	0	0	0	0	0	0	0	0	0	0	0	0	0	0	
	Upper limb	0	0	0	0	0	0	0	0	0	0	0	0	0	0	0	0	0	0	0	0	0	0	0	0	0	
	Lower limb	0	0	0	0	0	0	0	0	0	0	0	0	0	0	0	0	0	0	0	0	0	0	1	0	1	
	Multisite	0	0	0	0	0	0	0	0	0	0	0	0	0	0	0	0	0	0	0	0	0	0	0	0	0	
Degenerative joint disease	TMJ, head	0	0	0	0	0	0	0	0	0	0	0	0	0	0	0	0	0	0	0	0	0	0	0	0	0	10
	Back, neck, and pelvis	0	0	4	0	0	0	0	0	0	0	0	0	0	0	0	0	0	0	0	0	0	0	3	0	7	
	Upper limb	0	0	0	0	0	0	0	0	0	0	0	0	0	0	0	0	0	0	0	0	0	0	0	0	0	
	Lower limb	0	0	0	0	0	0	0	0	0	0	0	0	0	0	0	0	0	0	0	0	1	1	1	0	3	
	Multisite	0	0	0	0	0	0	0	0	0	0	0	0	0	0	0	0	0	0	0	0	0	0	0	0	0	
Rheumatic inflammatory disorders	TMJ, head	0	0	0	0	0	0	0	0	0	0	0	0	0	0	0	0	0	0	0	0	0	0	0	0	0	5
	Back, neck, and pelvis	0	0	1	0	0	0	0	0	0	0	0	0	0	0	0	0	0	0	0	0	0	0	0	0	1	
	Upper limb	0	0	0	0	0	0	0	0	0	0	0	0	0	0	0	0	0	0	0	0	0	0	0	0	0	
	Lower limb	0	0	0	0	0	0	0	0	0	0	0	0	0	0	0	0	0	0	0	0	0	0	0	0	0	
	Multisite	0	0	0	0	0	0	0	0	0	0	0	2	0	0	0	0	0	0	2	0	0	0	0	0	4	
Bone health and osteoporosis	TMJ, head	0	0	0	0	0	0	0	0	0	0	0	0	0	0	0	0	0	0	0	0	0	0	0	0	0	0
	Back, neck, and pelvis	0	0	0	0	0	0	0	0	0	0	0	0	0	0	0	0	0	0	0	0	0	0	0	0	0	
	Upper limb	0	0	0	0	0	0	0	0	0	0	0	0	0	0	0	0	0	0	0	0	0	0	0	0	0	
	Lower limb	0	0	0	0	0	0	0	0	0	0	0	0	0	0	0	0	0	0	0	0	0	0	0	0	0	
	Multisite	0	0	0	0	0	0	0	0	0	0	0	0	0	0	0	0	0	0	0	0	0	0	0	0	0	
Fracture	TMJ, headache	0	0	0	0	0	0	0	0	0	0	0	0	0	0	0	0	0	0	0	0	0	0	0	0	0	2
	Back, neck, and pelvis	0	0	0	0	0	0	0	0	0	0	0	0	0	0	0	0	0	0	0	0	0	0	0	0	0	
	Upper limb	0	0	0	0	0	0	0	0	0	0	0	0	0	0	0	0	0	0	0	0	0	0	0	0	0	
	Lower limb	0	0	0	0	0	0	0	0	0	0	0	0	0	0	0	0	0	1	0	0	0	0	0	0	1	
	Multisite	0	0	0	0	0	0	0	0	0	0	0	0	0	0	1	0	0	0	0	0	0	0	0	0	1	
Other	TMJ, headache	0	0	0	0	0	0	0	0	0	0	0	0	0	0	0	0	0	0	0	0	0	0	0	0	0	2
	Back, neck, and pelvis	0	0	0	0	0	0	0	0	0	0	0	0	0	0	0	0	0	0	0	0	0	0	0	0	0	
	Upper limb	0	0	0	0	0	0	0	0	0	0	0	0	0	0	0	0	0	0	0	0	0	0	0	0	0	
	Lower limb	0	0	0	0	0	0	0	0	0	0	0	0	0	0	0	0	0	0	0	0	0	0	0	0	0	
	Multisite	0	0	2	0	0	0	0	0	0	0	0	0	0	0	0	0	0	0	0	0	0	0	0	0	2	
	Subtotal	0	7	25	1	0	0	0	0	8	0	6	2	0	0	1	0	0	1	2	0	1	1	5	0		
	Total	33				0				16				1				3				7					60

RCT = Randomized controlled trial, CC = Case–control study, C = Cohort study, Q = Qualitative study, TMJ = Temporomandibular joint.

### Funding received for MSK disorder-related research through first-tier Swedish national competitive grants 2010–2020 registered in SweCRIS

As displayed in Table 4, research on rheumatic inflammatory disorders most frequently received funding through first-tier grants (127/305, 42%) amounting to 585 million Swedish kronor (MSEK), which is 50% of the total amount of 1,182 MSEK received by MSK disorder-related research. Otherwise, pain disorder-related grants (54/305, 18%) amounted to 162 MSEK (14%); degenerative joint disorder-related grants (37/305, 12%) amounted to 117 MSEK (10%); bone health and osteoporosis-related grants (33/305, 11%) amounted to 151 MSEK (13%); fracture-related grants (31/305,10%) amounted to 119 MSEK (10%), while joint, tendon, and muscle-related grants (13/305, 4%) as well as other diverse MSK disorder research programs (10/305, 3%) amounted to 30 MSEK (3%) and 23 MSEK (2%) of the total funding, respectively. Publications on diagnosis, prognosis, and healthcare organizational-related interventions (150/305, 49%) as well as pharmacological interventions (60/305, 20%) attracted 645 MSEK (55%) and 237 MSEK (20%) of the total research funds, respectively. Of these, the largest proportion was on rheumatic inflammatory disorders.

**Table 4:** First-tier funding grants for musculoskeletal disorder-related research through Swedish national competitive grants 2010–2020.

Diagnostic category	Diagnosis, prognosis, and healthcare organizational-related interventions		Preventative interventions		Rehabilitation interventions		Environmental modification, ADL aids, and physical modalities		Pharmacological interventions		Surgery and other orthopedic-related interventions		Other		Total	
	Funding MSEK	N	Funding MSEK	N	Funding MSEK	N	Funding MSEK	N	Funding MSEK	N	Funding MSEK	N	Funding MSEK	N	Funding MSEK	N
Pain disorders	79.37	28	14.29	4	64.95	16	1.97	2	1.66	4	0	0	0	0	162.24	54
Joint, tendon, and muscle injuries	6.10	2	5.10	2	12.40	4	0	0	0	0	6.24	5	0	0	29.84	13
Degenerative joint disease	55.58	12	5.18	1	9.27	8	0	0	2.00	2	45.16	14	0	0	117.19	37
Rheumatic inflammatory disorders	377.79	76	4.80	1	5.00	3	0	0	197.30	47	0	0	0	0	584.89	127
Bone health and osteoporosis	87.27	19	59.90	13	0	0	0	0	3.5	1	0	0	0	0	150.67	33
Fracture	32.73	9	0	0	0	0	22.68	4	26.60	5	37.00	13	0	0	119.01	31
Other	6.32	4	0	0	5.48	2	5.00	1	5.00	1	0.17	1	1.20	1	23.17	10
Total	645.16	150	89.27	21	97.10	33	29.65	7	236.67	60	82.97	33	1.20	1	1,182.02	305

MSEK = Million Swedish Kronor, *N* = Number of research grants.

### Summary of the main results

In summary, the study included 3,025 publications from 479 Swedish affiliated authors. Primary health care was the basis for 14% of the publications, 84% from secondary health care, and 2% from occupational health care with a similar proportional distribution of first-tier research grant financing. Approximately one in six publications were RCTs, while the majority were of observational cohort design. Most publications in primary and occupational health care were related to pain disorders (51 and 67%), and in secondary health care, rheumatic inflammatory disorder-related publications were most prevalent (30%).

## Discussion

Scoping review of published MSK disorder research with Swedish affiliation demonstrates similar publication volume in the primary and secondary healthcare contexts regarding pain disorders, while other diagnostic categories for MSK disorder research are predominantly secondary health care based. In primary and occupational health care, most publications were related to first-line diagnostic and rehabilitation interventions for pain disorders, especially for back and neck pain and to a smaller extent joint, tendon, and muscle injuries as well as degenerative joint disorders. This distribution is motivated considering the extent of prevalence and burden of these conditions (1) which is a major consideration in identifying research priorities (19). Furthermore, MSK-related pain is one of the most common reasons for patients consulting a physiotherapist (89%) or general practitioner (20–40%) in primary care and is a common symptom for most MSK disorders (20–22).

Only 21% of the Swedish-affiliated primary healthcare research publication on MSK disorders 2010–2020 has applied RCT designs and few with placebo controls and hybrid effectiveness-implementation designs. In descriptive contrast to secondary care, large-scale cohort studies in primary care have been less frequent, and national quality register infrastructure development in primary health care has been slow. In primary care, healthcare process indicators and patient-rated outcomes are currently only collected for osteoarthritis, and the limited number of primary healthcare clinics providing multimodal team treatment of complex chronic pain conditions (23, 24) (Appendix 2). A primary healthcare quality registry is under development but currently only contains limited process indicator data for osteoarthritis but no other MSK disorders (25). Policy makers should, therefore, prioritize that these primary care national registries should capture a broader scope of MSK disorder data to help ensure the quality of primary care and support future research.

MSK pain disorder research in Swedish secondary health care has had a large focus on cohort studies, especially on the diagnosis, prognosis, and clinical course of chronic pain conditions. However, the use of RCT designs has been increasing in frequency regarding rehabilitation for complex chronic conditions. The national quality register for pain rehabilitation in secondary care has developed with good national coverage and completeness and has future potential for supporting register-based RCTs. MSK pain disorder research has received 18% of first-tier grants, and future research aiming to improve the quality of care and improve patient functioning, activity, and self-care ability should continue as high priority for researchers, funding bodies, and policymakers.

Publications concerning preventative interventions were relatively few in primary (10%), occupational (0%), and secondary (<1%) healthcare systems, despite this being an integral part of especially primary and occupational health care (26, 27). Furthermore, only 7% of first-tier research funding went toward preventative interventions 2010–2020. These preventative interventions were mainly related to bone health and osteoporosis, acute injuries, and falls/fracture prevention in the elderly. Healthcare consumer literature suggests that 90% of patients consulting a physiotherapist in primary care consider both preventative and treatment interventions important (22). MSK disorders among Europe’s working-age population have a large economic burden, and productivity may be further undermined because of increasing chronic illness in an ageing population with increasing retiring age (2). Furthermore, screening and treating osteoporosis to prevent fractures is also of importance in an ageing population (28). Preventative interventions should, therefore, be a higher priority for researchers, funding bodies, policymakers, and clinicians aiming to implement evidence-based interventions in order to prevent MSK injuries, reduce chronicity, and improve patient self-care ability.

A total of 21% of primary healthcare research publications, 13% of secondary care publications, and small proportion of first-tier funding (3%) have been based on joint, tendon, and muscle injuries. In Sweden in 2009, the total population incidence of healthcare consultations for injuries such as joint distortions, joint luxation, and tendon and muscle injuries was 8.3/1,000, 1.6/1,000, and 1.8/1,000 population, respectively, with highest proportion of injuries in the younger population (29). Databases covering claims from compulsory insurance for sporting injuries suggest a national incidence for all types of sports and knee injuries of 10.2/1,000 athlete years (30). A large proportion of research publications have focused on knee injuries such as surgical treatment of anterior cruciate ligament (ACL) rupture and meniscal and/or cartilage damage, while achilles tendon ruptures have also been an area of interest. Over 50,000 primary ACL reconstructive surgeries have been reported in Swedish national knee ligament registry, which has good national coverage (Appendix 2). The registry is aiming for future inclusion of non-surgical intervention data collection as well as infrastructure for register RCT-based data collection (31). Despite the high volume of research activity, only few Swedish RCTs have compared effectiveness of surgical and rehabilitative interventions as well as attempted placebo-controlled designs. Improved registry infrastructure may aid better feasibility of conducting such research to improve quality of care.

Degenerative joint disorders including osteoarthritis and degenerative spinal conditions have attracted 12% of first-tier grants and produced 25% of secondary care publications. The prevalence of symptomatic degenerative joint disorders increases with age affecting up to one-third of the population and are ranked 11th among the leading causes of disability in the world (1). Biomarkers for the development of degenerative joint disorders have been a prevalent research theme in cohort studies. Surgical interventions have been the most prevalent interventions in cohort and RCT publications, which is reflected in the high surgery rates for degenerative joint disorders in Sweden and internationally (32). Again, only few Swedish RCTs have compared effectiveness of surgical and rehabilitative interventions as well as attempted placebo-controlled designs. Hip and knee arthroplasty as well as the spinal surgery national quality registries in Sweden provide good coverage and satisfactory completeness (Appendix 2), which could be utilized in the future to conduct national register RCTs rather than the current smaller single and multi-center ongoing RCTs that have received first-tier funding.

Research on fractures has produced 16% of secondary healthcare publications and 10% of first-tier grants, especially on diagnostic as well as surgical and other orthopedic-related interventions. In Sweden in 2009, the total population incidence of healthcare consultations for fractures was 18/1,000 population (29). Injuries due to falls have a high prevalence of disabling morbidity (1). The Swedish national fracture register records around 75,000 fractures each year that will continue to increase due to a greater coverage and completeness (Appendix 2). The register is the first in the MSK disorder field to conduct national register-based RCTs, providing a good example for future effectiveness research designs utilizing MSK-related national registers (33, 34). There are several problems regarding fracture treatment that need further research priority such as effectiveness and implementation studies concerning the management of upper extremity fractures among the elderly (35).

Compared to other MSK disorders, rheumatic and inflammatory disorders have a lower incidence (1.2/1,000 population) and burden of disease (1). Approximately 30% of Swedish-affiliated secondary healthcare research publications and approximately half of the first-tier funding have been in the field of rheumatic and inflammatory disorders. There has been a large investment in Sweden on translational research programs covering the etiology, pathogenesis, and potential treatment mechanisms. Collaboration with industry has also helped the development and clinical implementation of disease-modifying antirheumatic drugs, which have been proven efficacious through large multicenter placebo controlled RCTs (36). The Swedish rheumatic quality register (SRQ) contains around 80,000 unique patients with high national coverage and satisfactory data completeness (Appendix 2). This provides strong infrastructure for monitoring the quality of care, aiding the implementation of knowledge into clinical practice and the future possibility of performing national register-based RCT data collections.

To support the development of future register-based RCTs, Swedish guidelines have been developed (37). Use of the recently developed DITTO framework may help support the design of invasive placebo interventions, and recommendations for placebo-controlled trials in the rehabilitation field may help the planning of future high-quality RCT studies (38, 39). Resource efficient hybrid designs for RCTs involving both effectiveness and implementation process evaluations are also attracting more attention to accelerate the translation of the research findings to real world practice (40).

There are several strengths and weaknesses one must consider in the interpretation of the current study’s results. A strength of the methods is the use of a broad search criterion including MESH terms and relevant synonyms applied to relevant databases to capture the scope of recently published Swedish-affiliated MSK disorder researchers. The study selection process also involved independent reviewers and consensus meetings to solve ambiguities. This sampling procedure provided valid scope to aid future development of a national MSK clinical trial network in Sweden (SweMSK). This was followed by a process of database searches capturing the past 10 years of publications from this sample of recently published authors, and a manual study selection process was performed in a controlled manner by the review team. One must, however, keep in mind that the resulting scope of MSK research does not fully capture publications from previous authors who did have not recent publication activity.

## Conclusions

There has been strong tradition in Swedish-affiliated MSK disorder research 2010–2020 regarding observational cohort research but substantially less RCT research and also skewed first-tier funding allocation considering prevalence/incidence and burden of disease. A larger focus on prevention and clinical intervention-based RCT research in primary and occupational health care may be warranted. Use of infrastructure supporting register-based RCTs, placebo-controlled RCTs, and hybrid effectiveness-implementation studies is important strategies for the future for primary, occupational, and secondary care.

## Disclosure statement

LD is the co-founder and Chief Medical Officer of Joint Academy, a company that provides digital first-line intervention for patients with hip and knee osteoarthritis. The other authors declare that they have no conflicts of interest.

## Funding

This study was financially supported by the Swedish Research Council, grant number (DNR 2019-06101).

## Notes on contributors

***Elias Diarbakerli***, MSc, PhD, is a Physiotherapist and researcher at Karolinska University Hospital and the Department of Clinical Sciences, Intervention and Technology (CLINTEC), Karolinska Institutet, Stockholm, Sweden.

***Olof Thoreson***, MD, PhD is a General Practitioner and researcher at the Sahlgrenska Academy, University of Gothenburg, Sweden.

***Martin Björklund***, MSc, PhD, is a Physiotherapist and Associate Professor at the Department of Community Medicine and Rehabilitation, Umeå University; and the Centre for Musculoskeletal Research, Department of Occupational Health Sciences and Psychology, University of Gävle, Sweden.

***Leif Dahlberg***, MD, PhD, an Orthopedic Consultant and Senior Professor at the Department of Clinical Sciences Lund, Orthopaedics, Lund University, Sweden.

***Martin Englund***, MD, PhD, is an Epidemiologist and Professor at the Department of Clinical Sciences Lund, Orthopaedics, Lund University, Sweden.

***Paul Gerdhem***, MD, PhD, is an Orthopedic Consultant and Senior Professor at Karolinska University Hospital and the Department of Clinical Sciences, Intervention and Technology (CLINTEC), Karolinska Institutet, Stockholm, Sweden.

***Joanna Kvist***, MSc, PhD, is a Physiotherapist and Professor at Stockholm Sports Trauma Research Center, Karolinska Institute, Sweden; and the Department of Health, Medicine and Caring Sciences, Linköping University, Sweden.

***Maziar Mohaddes***, MD, PhD, an Orthopedic Consultant and Senior Professor at the Department of Orthopaedics, Institute of Clinical Sciences, Sahlgrenska Academy, University of Gothenburg, and Sahlgrenska University Hospital, Sweden.

***Anneli Peolsson***, MSc, PhD, is a Physiotherapist and Professor at the Department of Health, Medicine and Caring Sciences, Linköping University, and Occupational and Environmental Medicine Center, Linköping University, Sweden.

***Ola Rolfson***, MD, PhD, is an Orthopedic Consultant and Senior Professor at the Department of Orthopaedics, Institute of Clinical Sciences, Sahlgrenska Academy, University of Gothenburg, and Sahlgrenska University Hospital, Sweden.

***Birgitta Öberg***, MSc, PhD, is a Physiotherapist and Senior Professor at the Department of Health, Medicine and Caring Sciences, Linköping University, and Occupational and Environmental Medicine Center, Linköping University, Sweden.

***Allan Abbott***, MSc, PhD, is a Physiotherapist and Associate Professor at the Department of Health, Medicine and Caring Sciences, Linköping University, and Department of Orthopaedics, Linköping University Hospital, Sweden.

## ORCID

Elias Diarbakerli https://orcid.org/0000-0002-4511-4349

Olof Thoreson https://orcid.org/0000-0002-0978-541X

Martin Björklund https://orcid.org/0000-0001-7543-4397

Leif E Dahlberg https://orcid.org/0000-0001-7838-9464

Martin Englund https://orcid.org/0000-0003-3320-2437

Paul Gerdhem https://orcid.org/0000-0001-8061-7163

Joanna Kvist https://orcid.org/0000-0003-3527-5488

Maziar Mohaddes https://orcid.org/0000-0003-1848-9054

Anneli Peolsson https://orcid.org/0000-0002-6075-4432

Ola Rolfson https://orcid.org/0000-0001-6534-1242

Birgitta Öberg https://orcid.org/0000-0001-8612-583X

Allan Abbott https://orcid.org/0000-0002-4318-9216

## Appendix

**Appendix 1:** Search strategy.

Database	Search terms
PubMed 2021-01-18	(Sweden[affiliation]) and ((Low back pain[title/abstract]) OR (Back pain[Title/Abstract]) OR (Neck pain[Title/Abstract]) OR (Shoulder pain[Title/Abstract]) OR (subacromial pain[Title/Abstract]) OR (Elbow pain[Title/Abstract]) OR (Wrist pain[Title/Abstract]) OR (Hand pain[Title/Abstract]) OR (Pelvic Pain[Title/Abstract]) OR (Sacroiliac pain[Title/Abstract]) OR (Hip pain[Title/Abstract]) OR (Knee pain[Title/Abstract]) OR (Patellofemoral pain[Title/Abstract]) OR (Ankle pain[Title/Abstract]) OR (Foot pain[Title/Abstract]) OR (Arm pain[Title/Abstract]) OR (Leg pain[Title/Abstract]) OR (Sciatica[Title/Abstract]) OR (Radiculopathy[Title/Abstract]) OR (Impingement[Title/Abstract]) OR (Musculoskeletal pain[Title/Abstract]) OR (Muscle pain[Title/Abstract]) OR (Joint pain[Title/Abstract]) OR (Nociceptive pain[Title/Abstract]) OR (Disc herniation[Title/Abstract]) OR (Spondylolisthesis[Title/Abstract]) OR (Disc disease[Title/Abstract]) OR (Spinal stenosis[Title/Abstract]) OR (Osteoarthritis[Title/Abstract]) OR (Osteoporosis[Title/Abstract]) OR (Rheumatoid arthritis[Title/Abstract]) OR (Psoriatic arthritis[Title/Abstract]) OR (ankylosing spondylitis[Title/Abstract]) OR (Myalgia[Title/Abstract]) OR (Fibromyalgia[Title/Abstract]) OR (injury[Title/abstract]) OR (Fracture[Title/Abstract]) OR (Rupture[Title/Abstract]) OR (Tear[Title/Abstract]) OR (Whiplash [Title/Abstract]) OR (WAD[Title/Abstract])) NOT ((Cancer[Title/Abstract]) OR (Cardiac[Title/Abstract]) OR (Coronary[Title/Abstract]) OR (Artery[Title/Abstract]) OR (Ischemic[Title/Abstract]) OR (Perfusion[Title/Abstract]) OR (Infarct*[Title/Abstract]) OR (Stroke[Title/Abstract]) OR (Brain[Title/Abstract]) OR (Aneurysm[Title/Abstract]) OR (Diabetes[Title/Abstract]) OR (Nerv*[Title/Abstract]) OR (Neurological[Title/Abstract]) OR (Cord[Title/Abstract]) OR (Head[Title/Abstract]) OR (Lung[Title/Abstract]) OR (Kidney[Title/Abstract]) OR (Renal[Title/Abstract]) OR (Liver[Title/Abstract]) OR (Biliary[Title/Abstract]) OR (Bile[Title/Abstract]) OR (Cholangitis[Title/Abstract]) OR (Cataract[Title/Abstract]) OR (Labour [Title/Abstract]) OR (Obstetric[Title/Abstract]) OR (Allergi*[Title/Abstract]) OR (Vaccine[Title/Abstract]) OR (Burn[Title/Abstract]) OR (Endothelial[Title/Abstract]) OR (Self-injury[Title/Abstract]) OR (Violence[Title/Abstract]) OR (Syndrome[Title/Abstract]))
Scopus 2021-01-18	TITLE-ABS-KEY ( low AND back AND pain ) OR TITLE-ABS-KEY ( back AND pain ) OR TITLE-ABS-KEY ( neck AND pain ) OR TITLE-ABS-KEY ( shoulder AND pain ) OR TITLE-ABS-KEY ( subacromial AND pain ) OR TITLE-ABS-KEY ( elbow AND pain ) OR TITLE-ABS-KEY ( wrist AND pain ) OR TITLE-ABS-KEY ( hand AND pain ) OR TITLE-ABS-KEY ( pelvic AND pain ) OR TITLE-ABS-KEY ( sacroiliac AND pain ) OR TITLE-ABS-KEY ( hip AND pain ) OR TITLE-ABS-KEY ( knee AND pain ) OR TITLE-ABS-KEY ( patellofemoral AND pain ) OR TITLE-ABS-KEY ( ankle AND pain ) OR TITLE-ABS-KEY ( foot AND pain ) OR TITLE-ABS-KEY ( arm AND pain ) OR TITLE-ABS-KEY ( leg AND pain ) OR TITLE-ABS-KEY ( sciatica ) OR TITLE-ABS-KEY ( radiculopathy ) OR TITLE-ABS-KEY ( impingement ) OR TITLE-ABS-KEY ( musculoskeletal AND pain ) OR TITLE-ABS-KEY ( muscle AND pain ) OR TITLE-ABS-KEY ( joint AND pain ) OR TITLE-ABS-KEY ( nociceptive AND pain ) OR TITLE-ABS-KEY ( disc AND herniation ) OR TITLE-ABS-KEY ( spondylolisthesis ) OR TITLE-ABS-KEY ( disc AND disease ) OR TITLE-ABS-KEY ( spinal AND stenosis ) OR TITLE-ABS-KEY ( osteoarthritis ) OR TITLE-ABS-KEY ( osteoporosis ) OR TITLE-ABS-KEY ( rheumatoid AND arthritis ) OR TITLE-ABS-KEY ( psoriatic AND arthritis ) OR TITLE-ABS-KEY ( ankylosing AND spondylitis ) OR TITLE-ABS-KEY ( myalgia ) OR TITLE-ABS-KEY ( fibromyalgia ) OR TITLE-ABS-KEY ( injury ) OR TITLE-ABS-KEY ( fracture ) OR title-abs- KEY ( rupture ) OR TITLE-ABS-KEY ( tear ) OR TITLE-ABS-KEY ( whiplash ) OR TITLE-ABS-KEY ( wad ) AND AFFIL ( sweden ) AND NOT TITLE-ABS-KEY ( cancer ) OR TITLE-ABS-KEY ( cardiac ) OR TITLE-ABS-KEY ( coronary ) OR TITLE-ABS-KEY ( artery ) OR TITLE-ABS-KEY ( ischemic ) OR TITLE-ABS-KEY ( perfusion ) OR TITLE-ABS-KEY ( infarct* ) OR TITLE-ABS-KEY ( stroke ) OR TITLE-ABS-KEY ( brain ) OR TITLE-ABS-KEY ( aneurysm ) OR TITLE-ABS-KEY ( diabetes ) OR TITLE-ABS-KEY ( nerv* ) OR TITLE-ABS-KEY ( neurological ) OR TITLE-ABS-KEY ( cord ) OR TITLE-ABS-KEY ( head ) OR TITLE-ABS-KEY ( lung ) OR TITLE-ABS-KEY ( kidney ) OR TITLE-ABS-KEY ( renal ) OR TITLE-ABS-KEY ( liver ) OR TITLE-ABS-KEY ( biliary ) OR TITLE-ABS-KEY ( bile ) OR TITLE-ABS-KEY ( cholangitis ) OR TITLE-ABS-KEY ( cataract ) OR TITLE-ABS-KEY ( labour ) OR TITLE-ABS-KEY ( obstetric ) OR TITLE-ABS-KEY ( allergi* ) OR TITLE-ABS-KEY ( vaccine ) OR TITLE-ABS-KEY ( burn ) OR TITLE-ABS-KEY ( endothelial ) OR TITLE-ABS-KEY ( self-injury ) OR TITLE-ABS-KEY ( violence ) OR TITLE-ABS-KEY ( syndrome ) AND ( LIMIT-TO ( DOCTYPE , “ar” ) ) AND ( LIMIT-TO ( SUBJAREA , “MEDI” ) OR LIMIT-TO ( SUBJAREA , “HEAL” ) ) AND ( LIMIT-TO ( SRCTYPE , “j” ) )
PubMed 2021	Publications of each of the 479 authors were individually searched, and the Boolean operator ‘NOT’ was used to filter out publications which included the other authors. For example: (Anders Ekbbom[Author]) NOT ((Anna Rudin[Author]) OR (Jon Lampa[Author]) OR (Joakim Lindqvist[Author]) OR (Meliha Kapetanovic[Author]) OR (Giovanni Cagnotto[Author]) OR (Eva Baecklund[Author]) OR (Tomas Husmark[Author]) OR (Åsa Reckner-Olsson[Author]) OR (Per Larsson[Author]) OR (Asgeir Jakola[Author]) OR (Mattias Lorentzon[Author]) OR (Liesbeth Vandenput[Author]) OR (Christina Kaijser Alin[Author]) OR (Nathalie Frisendahl[Author]) OR (Ann-Charlotte Grahn Kronhed[Author]) OR (Helena Salminen[Author]) OR (Olof Wolf[Author]) OR (Sebastian Mukka[Author]) OR (Jan Ekelund[Author]) OR (Michael Möller[Author]) OR (Nils P Hailer[Author]) OR (Anneli Peolsson[Author]) OR (Johanna Wibault[Author]) OR (Håkan Löfgren[Author]) OR (Åsa Dedering[Author]) OR (Peter Zsigmond[Author]) OR (Åsa Karlsson[Author]) OR (Monica Berggren[Author]) OR (Birgitta Olofsson[Author]) OR (Michael Stenvall[Author]) OR (Yngve Gustafson[Author]) OR (Peter Nordström[Author]) OR (Nina Lindelöf[Author]) OR (Aleksandra Turkiewicz[Author]) OR (Martin Englund[Author]) OR (Gunnar Flivik[Author]) OR (Gösta Ullmark[Author]) OR (Jens Sörensen[Author]) OR (Olle Nilsson[Author]) OR (Anna Marseglia[Author]) OR (Anna Hermansen[Author]) OR (Birgitta Öberg[Author]) OR (Paul Enthoven[Author]) OR (Anna Spångeus[Author]) OR (Mikko Juhani Lammi[Author]) OR (Åsa Degerstedt[Author]) OR (Hassan Alinaghizadeh[Author]) OR (Carina Thorstensson[Author]) OR (Christina B Olsson[Author]) OR (Lisa Theander[Author]) OR (Minna Willim[Author]) OR (Jan Åke Nilsson[Author]) OR (Magnus Karlsson[Author]) OR (Kristina Åkesson[Author]) OR (Lennart Jacobsson[Author]) OR (Carl Turesson[Author]) OR (Christina Constantinou[Author]) OR (Ninni Sernert[Author]) OR (Lars Rostgård-Christensen[Author]) OR (Juri Kartus[Author]) OR (Per Jolbäck[Author]) OR (Emma Nauclér[Author]) OR (Erik Bülow[Author]) OR (Hans Lindahl[Author]) OR (Maziar Mohaddes[Author]) OR (Karin Schröder[Author]) OR (Allan Abbott[Author]) OR (Philip Moons[Author]) OR (Andreas Eklund[Author]) OR (Jan Hagberg[Author]) OR (Irene Jensen[Author]) OR (Peter Lövgren[Author]) OR (Iben Axén[Author]) OR (Emma Söreskog[Author]) OR (Fredrik Borgström[Author]) OR (Helena Johansson[Author]) OR (Erland Axelsson[Author]) OR (Maria Hedman-Lagerlöf[Author]) OR (Erik Hedman-Lagerlöf[Author]) OR (Brjánn Ljótsson[Author]) OR (Evelina Blomqwist[Author]) OR (Cecilia Rogmark[Author]) OR (Glenn Larsson[Author]) OR (Anna Nilsdotter[Author]) OR (Sofie Ahlen[Author]) OR (Ingrid Larsson[Author]) OR (Stefan Lohmander[Author]) OR (Urban Berg[Author]) OR (Annette W-Dahl[Author]) OR (Ola Rolfson[Author]) OR (Martin Sundberg[Author]) OR (Andreas Älgå[Author]) OR (Johan von Schreeb[Author]) OR (Jonas Malmstedt[Author]) OR (Anna K E Vadell[Author]) OR (Erik Hulander[Author]) OR (Inger Gjertsson[Author]) OR (Helen M Lindqvist[Author]) OR (Anna Winkvist[Author]) OR (Johanna Rundgren[Author]) OR (Alicja Bojan[Author]) OR (Cecilia Mellstrand-Navarro[Author]) OR (Anders Enocson[Author]) OR (Pär Wennberg[Author]) OR (Margareta Möller[Author]) OR (Elisabeth Kenne Sarenmalm[Author]) OR (Johan Herlitz[Author]) OR (Malin Forsbrand[Author]) OR (Ingemar Petersson[Author]) OR (Charlotte Post Sennehed[Author]) OR (Kjerstin Stigmar[Author]) OR (Stefano Romeo[Author]) OR (Daniel Glinatsi[Author]) OR (Ida Åkerlund[Author]) OR (Markus Waldén[Author]) OR (Sofi Sonesson[Author]) OR (Martin Hägglund[Author]) OR (Ulrika Aasa[Author]) OR (Lars Berglund[Author]) OR (Michele Compagno[Author]) OR (Saedis Saevarsdottir[Author]) OR (Johan Askling[Author]) OR (Annelie Bilberg[Author]) OR (Birgitta Grahn[Author]) OR (Marcelo Rivano Fischer[Author]) OR (Anja Nyberg[Author]) OR (Sara Holmberg[Author]) OR (Mats Ranebo[Author]) OR (Hanna Björnsson Hallgren[Author]) OR (Theresa
	Holmgren[Author]) OR (Lars Adolfsson[Author]) OR (Kristin Gustafsson[Author]) OR (Joanna Kvist[Author]) OR (Marit Eriksson[Author]) OR (Susanne Hansson[Author]) OR (Anne Garland[Author]) OR (Henrik Malchau[Author]) OR (Susanna Aufwerber[Author]) OR (Gunnar Edman[Author]) OR (Paul Ackermann[Author]) OR (Barbara A Snoeker[Author]) OR (Anna Svärd[Author]) OR (Mikeal Brink[Author]) OR (Christopher Sjöwall[Author]) OR (Solbritt Rantapää Dahlqvist[Author]) OR (Alf Kastbom[Author]) OR (Nancy Pedersen[Author]) OR (Eleonor Svantesson[Author]) OR (Eric Hamrin Senorski[Author]) OR (Ferid Krupic[Author]) OR (Olof Westin[Author]) OR (Kristian Samuelsson[Author]) OR (Robert Dutkiewicz[Author]) OR (Bengt Nellgård[Author]) OR (Karin Roos Ljungberg[Author]) OR (Michael Ziegelasch[Author]) OR (Jan Cedergren[Author]) OR (Per Eriksson[Author]) OR (Åsa Reckner[Author]) OR (Peter Svensson[Author]) OR (Andrea Dell’Isola[Author]) OR (Thérése Jönsson[Author]) OR (Jonas Ranstam[Author]) OR (Leif E Dahlberg[Author]) OR (Eva Ekvall Hansson[Author]) OR (Marie Kierkegaard[Author]) OR (Paula Kelly-Pettersson[Author]) OR (Bodil Samuelsson[Author]) OR (Maria Unbeck[Author]) OR (Olav Muren[Author]) OR (Martin Magnéli[Author]) OR (Max Gordon[Author]) OR (André Stark[Author]) OR (Olof Skoldenberg[Author]) OR (Vasileios Zampelis[Author]) OR (Uldis Kesteris[Author]) OR (Christoffer von Essen[Author]) OR (Sebastian McCallum[Author]) OR (Björn Barenius[Author]) OR (Karl Eriksson[Author]) OR (Rune Hedlund[Author]) OR (Ann-Sofi Kammerlind[Author]) OR (Tobias Lagerbäck[Author]) OR (Hans Möller[Author]) OR (Paul Gerdhem[Author]) OR (Gunnel Peterson[Author]) OR (Georgios Chatziagorou[Author]) OR (Johan Kärrholm[Author]) OR (Emma Haglund[Author]) OR (Ann Bremander[Author]) OR (Stefan Bergman[Author]) OR (Bodil Halvarsson[Author]) OR (Philip Von Rosen[Author]) OR (Kaisa Mannerkorpi[Author]) OR (Frida Eek[Author]) OR (Kristine Kalland[Author]) OR (Henrik Åberg[Author]) OR (Michael Ullman[Author]) OR (Greta Snellman[Author]) OR (Kenneth B Jonsson[Author]) OR (Torsten Johansson[Author]) OR (Britt-Marie Nyhäll-Wåhlin[Author]) OR (Sofia Ajeganova[Author]) OR (Maria Andersson[Author]) OR (Jenny Liu[Author]) OR (Sari Ponzer[Author]) OR (Nasim Farrokhnia[Author]) OR (Peter Ström[Author]) OR (Hans E Berg[Author]) OR (Li Felländer-Tsai[Author]) OR (Karl-Åke Jansson[Author]) OR (Daniel Fell[Author]) OR (Chan-Mei Ho[Author]) OR (Lena Nordeman[Author]) OR (Karen Hambardzumyan[Author]) OR (Nancy Vivar[Author]) OR (Sofia Ernestam[Author]) OR (Johan Wallman[Author]) OR (Ronald van Vollenhoven[Author]) OR (Anna Fogdell-Hahn[Author]) OR (Lena W Holm[Author]) OR (Eva Skillgate[Author]) OR (Jenny Saving[Author]) OR (Maria Wilcke[Author]) OR (Jabbar Mohammed[Author]) OR (Carl-Johan Hedbeck[Author]) OR (Ghazi Chammout[Author]) OR (Linnea Oldsberg[Author]) OR (Ingrid Osika Friberg[Author]) OR (Anke Samulowitz[Author]) OR (Szilárd Nemes[Author]) OR (Ammar Jobory[Author]) OR (Inger Nilsson[Author]) OR (Filip Gedin[Author]) OR (Ann-Charlotte Egmar[Author]) OR (Tobias Sundberg[Author]) OR (Kristina Burström[Author]) OR (Christer Rolf[Author]) OR (Knut Aagaard[Author]) OR (Karl Lunsjö[Author]) OR (Ulla Lindqvist[Author]) OR (Hanna Lotzke[Author]) OR (Helena Brisby[Author]) OR (Annelie Gutke[Author]) OR (Olle Hägg[Author]) OR (Max Jakobsson[Author]) OR (Mari Lundberg[Author]) OR (Arkan Sayed-Noor[Author]) OR (Anna Nilsdotter[Author]) OR (Felix Cronholm[Author]) OR (Björn Rosengren[Author]) OR (Jan-Åke Nilsson[Author]) OR (Claes Ohlsson[Author]) OR (Dan Mellström[Author]) OR (Eva Ribom[Author]) OR (Tomas Weitoft[Author]) OR (Kajsa Öberg[Author]) OR (Annelie Bilberg[Author]) OR (Richard Frobell[Author]) OR (Linda Karlsson[Author]) OR (Anders Joelson[Author]) OR (Elias Diarbakerli[Author]) or (Per Wretenberg[Author]) or (Karin Frennered[Author]) OR (Anna MacDowall[Author]) OR (Nuno Canto Moreira[Author]) OR (Catarina Marques[Author]) OR (Martin Skeppholm[Author]) OR (Lars Lindhagen[Author]) OR (Yohan Robinson[Author]) OR (Karl Michaëlsson[Author]) OR (Claes Olerud[Author]) OR (David Conradsson[Author]) OR (Alexandra Halvarsson[Author]) OR (Elin Uzunel[Author]) OR (Martin Gerdin[Author]) OR (Lucy Law[Author]) OR (Jeanette Beckman Rehnman[Author]) OR (Anna Deminger[Author]) OR (Eva Klingberg[Author]) OR (Helena Forsblad-D’Elia[Author]) OR (Maura Iversen[Author]) OR (Monika Hansson[Author]) OR (Monika Löfgren[Author]) OR (Christina Opava[Author]) OR (Ingrid Demmelmaier[Author]) OR (Cecilia Fridén[Author]) OR (Ingrid Lundberg[Author]) OR (Birgitta Nordgren[Author]) OR (Eva Kosek[Author]) OR (Marie Halvorsen[Author]) OR (Mikael Svensson[Author]) OR (Harald Brismar[Author]) OR (Ola Hallert[Author]) OR (Urban Lindgren[Author]) OR (Carina Boström[Author]) OR (Maria Hagströmer[Author]) OR (Magnus H Jonsson[Author]) OR (Ami Hommel[Author]) OR (Ulf Ekelund[Author]) OR (Olle Melander[Author]) OR (Peter Bentzer[Author]) OR (Katerina Chatzidionysiou[Author]) OR (Sören Toksvig-Larsen[Author]) OR (Jan G Jakobsson[Author]) OR (Emilia Rydberg Moller[Author]) OR (Malin Lohela-Karlsson[Author]) OR (Lennart Bodin[Author]) OR (Sanjib Saha[Author]) OR (Ulf G Gerdtham[Author]) OR (Sara Holmberg[Author]) OR (Johan Jarl[Author]) OR (Jonas Wetterö[Author]) OR (Thomas Skogh[Author]) OR (Carl Johan Tiderius[Author]) OR (Ulf Lindström[Author]) OR (Undis Englund[Author]) OR (Christian Inngul[Author]) OR (Zlatan Alagic[Author]) OR (Johanna Gustavsson[Author]) OR (Carl Bonander[Author]) OR (Finn Nilson[Author]) OR (Magni Mohr[Author]) OR (Karin Magnusson[Author]) OR (Therese Jönsson[Author]) OR (Patrick Bergman[Author]) OR (Peter Axelsson[Author]) OR (Allan Ibsen Sörensen[Author]) OR (Fan Zhang[Author]) OR (Linda Mathsson Alm[Author]) OR (Pernilla Eliasson[Author]) OR (André Struglics[Author]) OR (Staffan Larsson[Author]) OR (Anna Pramhed[Author]) OR (Per Swärd[Author]) OR (Jonatan Attergrim[Author]) OR (Mattias Sterner[Author]) OR (Alice Claeson[Author]) OR (David Nilsson[Author]) OR (Johan Trygg[Author]) OR (Anna Nilsson[Author]) OR (Daniel Sundh[Author]) OR (Daniel Sundh[Author]) OR (Mahtaz Mojtaba[Author]) OR (Elisabeth Rydwik[Author]) OR (Jonas Holtenius[Author]) OR (Peyman Bakhshayesh[Author]) OR (Östen Ljunggren[Author]) OR (Tor Olofsson[Author]) OR (Anders Gülfe[Author]) OR (Jon Tjörnstrand[Author]) OR (Paul Neuman[Author]) OR (Björn Lundin[Author]) OR (Jonas Svensson[Author]) OR (Bijar Ghafouri[Author]) OR (Indre Bileviciute-Ljungar[Author]) OR (Jan Bjersing[Author]) OR (Annie Palstam[Author]) OR (Björn Gerdle[Author]) OR (Marie Askenberger[Author]) OR (Eva Moström[Author]) OR (Wilhelmina Ekström[Author]) OR (Christina Mikkelsen[Author]) OR (Per-Mats Janarv[Author]) OR (Elisabeth Svensson[Author]) OR (Bo Nyström[Author]) OR (Elvira Lange[Author]) OR (Sara Svedlund[Author]) OR (Karin Svensson[Author]) OR (Mikael Svensson[Author]) OR (Katarina Nilsson Helander[Author]) OR (Karin Grävare Silbernagel[Author]) OR (Nicklas Olsson[Author]) OR (Jón Karlsson[Author]) OR (Elisabeth Hansson Olofsson[Author]) OR (Hans O Thulesius[Author]) OR (Tove Eneljung[Author]) OR (Eling D de Bruin[Author]) OR (Ayako Hiyoshi[Author]) OR (Martin Nilsson[Author]) OR (Michail Zoulakis[Author]) OR (Mats Brittberg[Author]) OR (Åsa Svedmark[Author]) OR (Martin Björklund[Author]) OR (Charlotte K Häger[Author]) OR (Johan Nilsson Sommar[Author]) OR (Jens Wahlström[Author]) OR (Jon T Einarsson[Author]) OR (Tore Saxne[Author]) OR (Pierre Geborek[Author]) OR (Björn Alkner[Author]) OR (Erika Andersson[Author]) OR (Peter Fritzell[Author]) OR (Vala flosadottir[Author]) OR (Eva Ageberg[Author]) OR (Håkan Alfredson[Author]) OR (Christoph Spang[Author]) OR (Susanne Beischer[Author]) OR (Christoffer Thomeé[Author]) OR (Roland Thomeé[Author]) OR (Susanna Larsson[Author]) OR (Håkan Melhus[Author]) OR (Lotte Skytte Kröll[Author]) OR (Catharina Sjödahl Hammarlund[Author]) OR (Gunvor Gard[Author]) OR (Ylva Aurell[Author]) OR (Maria Andersson[Author]) OR (Kristina Forslind[Author]) OR (Yvonne Lindbäck[Author]) OR (Hans Tropp[Author]) OR (Axel Svedbom[Author]) OR (Oskar Ström[Author]) OR (Liselotte Persson[Author]) OR (David Sundemo[Author]) OR (Ove Törring[Author]) OR (Rikard K Wicksell[Author]) OR (Ida Flink[Author]) OR (Annelie Brorsson[Author]) OR (Gunnel Carlsson[Author]) OR (Lena Zidén[Author]) OR (Gunilla Kjellby-Wendt[Author]) OR (Erik von Oelreich[Author]) OR (Mikael Eriksson[Author]) OR (Andrea Discacciati[Author]) OR (Lovisa Strömmer[Author]) OR (Anders Oldner[Author]) OR (Emma Larsson[Author]) OR (Johan Dalén[Author]) OR (Adad Baranto[Author]) OR (Stefan Malmqvist[Author]) (Urban Johnson[Author]) OR (Albert Christersson[Author]) OR (Sune Larsson[Author]) OR (Bengt Sandén[Author]) OR (Otto Robertsson[Author]) OR (Ján Kuchálik[Author]) OR (Anders Magnuson[Author]) OR (Anders Lundin[Author]) OR (Anil Gupta[Author]) OR (Göran Garellick[Author]) OR (Ola Olsson[Author]) OR (Clare Ardern[Author]) OR (Kajsa Johansson[Author]) OR (Tian Cheng[Author]) OR (Olof Brattström[Author]))

**Appendix 2:** Swedish national quality registries in the MSK disorder field. Information from annual reports based on data up to 2020.

Databases (*n* = unique subjects)	Available data					National coverage	National completeness
	Q	P	I	T	Longitudinal PROM data (% patients with both baseline and follow-up)		
**Primary care databases**							
BOA – hip, knee, and hand arthritis (*n* = 139,080)	x		x	x	3 m (68%), 12 m (41%)	90%	70%
NRS – multidisciplinary pain rehabilitation (*n* = 2,161)	x	x		x	After rehab (92%), 12 m (70%)	–	–
**Secondary care orthopedic databases**							
Swedish hip arthroplasty registry (*n* = 400,708)	x	x	x	x	12 m (65%)	100%	98%
Swedish knee arthroplasty registry (*n* = 239,839)	x	x	x	x	12 m (65%)	100%	97%
Swedish shoulder and elbow surgery registry (*n* = 23,800)	x	x	x	x	12 m & 5 y (40%)	85%	95%
Swedish fracture registry (*n* = 667,746)	x	x	x	x	12 m (40%)	100%	65%
Swedish hip fracture registry (*n* = 320,000)	x	x	x	x	4 m (60%)	85%	79%
Swedish ankle surgery registry (*n* = 6,000)	x	x	x	x	6 m & 12 m (60%)	100%	100%
Swedish foot surgery registry (*n* = 20,000)	x	x	x	x	12 m (40%)	68%	50%
Swedish cruciate ligament surgery registry (*n* = 52,734)	x	x	x	x	12 m (50%), 24 m (50%), 5 y (40%), 10 y (40%)	90%	95%
Swedish amputations and prosthesis registry (*n* = 8,406)	x	x	x	x	6m, 12m, 24m (40%)	76%	74%
Swedish spinal surgery registry (*n* = 137,717)	x	x	x	x	12 m (75%), 24 m (67%), 5 y (55%)	95%	85%
Swedish hand surgery registry (*n* = 127,031)	x	x	x	x	3 m & 12 m (45%)	80%	70%
Swedish pediatric orthopedic registry (*n* = 2,700)	x	x	x	x	12 m (50%)	96%	87%
**Secondary care pain rehabilitation databases**							
NRS-registry – multidisciplinary team pain rehabilitation in secondary health care (*n* = 21,356)	x			x	After rehab (83%), 12 m (70%)	100%	80%
**Secondary care rheumatology databases**							
Swedish rheumatology register (*n* = 100,000)	x			x	6 m (88%) & 12 m (66%)	84%	85%

MSK = musculoskeletal, Q = questionnaires on patient and disease characteristics, P = physical examination; I = imaging (radiographs, MRI, CT, or ultrasound), T = treatment characteristics, m = months, y = years.
